# Hybrid Performance of an Immortalized F_2_ Rapeseed Population Is Driven by Additive, Dominance, and Epistatic Effects

**DOI:** 10.3389/fpls.2017.00815

**Published:** 2017-05-18

**Authors:** Peifa Liu, Yusheng Zhao, Guozheng Liu, Meng Wang, Dandan Hu, Jun Hu, Jinling Meng, Jochen C. Reif, Jun Zou

**Affiliations:** ^1^National Key Laboratory of Crop Genetic Improvement, Huazhong Agricultural UniversityWuhan, China; ^2^Department of Breeding Research, Leibniz Institute of Plant Genetics and Crop Plant Research (IPK)Stadt Seeland, Germany

**Keywords:** hybrid performance, genome-wide prediction, dominance effects, epistasis, rapeseed

## Abstract

Genomics-based prediction of hybrid performance promises to boost selection gain. The main goal of our study was to investigate the relevance of additive, dominance, and epistatic effects for determining hybrid seed yield in a biparental rapeseed population. We re-analyzed 60,000 SNP array and seed yield data points from an immortalized F_2_ population comprised of 318 hybrids and 180 parental lines by performing genome-wide QTL mapping and predictions in combination with five-fold cross-validation. Moreover, an additional set of 37 hybrids were genotyped and phenotyped in an independent environment. The decomposition of the phenotypic variance components and the cross-validated results of the QTL mapping and genome-wide predictions revealed that the hybrid performance in rapeseed was driven by a mix of additive, dominance, and epistatic effects. Interestingly, the genome-wide prediction accuracy in the additional 37 hybrids remained high when modeling exclusively additive effects but was severely reduced when dominance or epistatic effects were also included. This loss in accuracy was most likely caused by more pronounced interactions of environments with dominance and epistatic effects than with additive effects. Consequently, the development of robust hybrid prediction models, including dominance and epistatic effects, required much deeper phenotyping in multi-environmental trials.

## Introduction

Hybrid breeding is a promising approach to boost selection gain in crop improvement (Duvick, 2001; Kempe and Gils, 2011; Zhang et al., 2016). The establishment of hybrid breeding programs for rapeseed (*Brassica napus*, 2n = 38, AACC) resulted in an up to 30% increase in seed yield compared with open-pollinated cultivars (Brandt et al., 2007). Hybrid breeding in rapeseed profited strongly from the exploration of different hybrid seed production systems, such as the Polima cytoplasmic male sterility (CMS) (Fu et al., 1995), Ogura CMS (Brown et al., 2003), genic male sterility (Yan et al., 2016), and ecotype male sterility (Yu et al., 2015). As a consequence, hybrids replaced open-pollinated cultivars in most rapeseed growing regions (Fu, 2000).

One major challenge in hybrid breeding has been to identify superior single-crosses out of millions of potential hybrids (Bernardo, 1994). Genome-wide prediction is a powerful tool to solve this problem, even for quantitative traits (Zhao et al., 2015b). In genome-wide prediction, many markers are used, and their effects are estimated in populations that have been genotyped and phenotyped. The estimated marker effects are then applied to predict the performance of non-phenotyped hybrids based on their molecular marker profiles.

The potential of genome-wide prediction was investigated in rapeseed with a focus on general combining ability (GCA) effects as the additive component of the hybrid performance (Jan et al., 2016). The study was based on two testcross series of 475 spring-type lines and revealed moderate-to-high prediction accuracies for a number of important agronomic traits. The potential of genome-wide prediction of hybrid performance, i.e., additive/general and non-additive/specific combining ability (SCA) effects, has not been examined in rapeseed. Simulation studies revealed that the prediction accuracy of the hybrid performance can be increased by modeling dominance as one type of non-additive effect, but the magnitude of improvement strongly depended on the relevance of the variance of specific vs. combining ability effects (Technow et al., 2012). Analyses of experimental data in maize (Bernardo, 1994), rice (Wang et al., 2017), wheat (Zhao et al., 2015a), triticale (Gowda et al., 2013), and sunflower (Reif et al., 2013) corroborated this finding, with hybrid prediction accuracies being either similar or higher when fitting additive and dominance effects.

Genome-wide prediction approaches of the hybrid performance can also accommodate epistasis, i.e., interaction effects between genes (Xu et al., 2014). In particular, semiparametric reproducing kernel Hilbert space (RKHS) regression models or extended genomic best linear unbiased predictions (EG-BLUP) are computational efficient approaches to capture epistasis in genome-wide predictions (Jiang and Reif, 2015). Experimental studies in rice (Xu et al., 2014), wheat (Zhao et al., 2015a), and apple (Kumar et al., 2015) have shown no or only marginal improved prediction accuracies through modeling additive, dominance, and epistatic effects. This outcome contrasted with the results of genome-wide prediction studies focused on wheat inbred lines that reported an increase in accuracy when fitting data based on effects besides additive and epistatic effects (He et al., 2016a,b). Furthermore, several attempts have been made to dissect the genetic basis of heterosis for important agronomic traits in rapeseed using biparental populations (Radoev et al., 2008; Basunanda et al., 2010; Shi et al., 2011; Bu et al., 2015; Wen et al., 2015). All previous studies reported that dominance and epistatic effects contributed to heterosis. Thus, it was tempting to hypothesize that genome-wide prediction of hybrid performance in rapeseed was profiting from modeling additive, dominance, and epistatic effects.

In our study, we re-analyzed published rapeseed data, which were previously used to determine the genetic basis of heterosis (Shi et al., 2011). The data comprised phenotypic records for seed yield generated for the biparental TNDH doubled haploid population and the corresponding derived immortalized F_2_ population (TNRC-F_2_) (Shi et al., 2011). Moreover, the parental lines have been fingerprinted with a 60,000 SNP array (Zhang et al., 2016), and an additional 37 new RC-F_2_ crosses were genotyped and phenotyped in this study. The objectives of our study were to investigate the relevance of additive, dominance, and epistatic effects for determining hybrid seed yield using genome-wide association mapping and to examine the potential to increase the accuracy of hybrid prediction when considering additive, dominance, and epistatic effects.

## Materials and methods

### Plant materials and field trials

In our previous study, a doubled haploid population of 202 lines (TNDH) was developed by microspore culture from the F_1_ cross between Tapidor (European winter-type rapeseed cultivar) and Ningyou7 (Chinese semi-winter type rapeseed cultivar; Qiu et al., 2006). The 202 DH lines were used to generate an immortalized F_2_ hybrid population (TNRC-F_2_) with 404 single crosses. Each DH line served as a parent for single-cross hybrids (Shi et al., 2011). The lines of TNDH and hybrids of TNRC-F_2_ were evaluated with Tapidor and Ningyou7 at three different environments, i.e., year-location combinations, in China (Supplementary Table 1). Details of the field evaluation were published elsewhere (Shi et al., 2011). Briefly, the plot size was 3.0 m^2^ with a distance of 40 cm between rows and 25 cm between individuals. The average dry weight of the seeds was determined as Mg ha^−1^.

Furthermore, 37 genotypes were sampled as an independent validation population. The validation population included 37 single-cross hybrids not considered in the TNRC-F_2_ population and were derived from the crosses among two lines of the 180 DH lines and 18 new DH lines derived from the TN (Tapidor × Ningyou7) F_1_ cross. The validation population was grown in one environment (2015–2016) in Wuhan in a trial with three replicates. Every plot comprised three rows with a total plot size of 1.8 m^2^. The average dry weight of the seeds was determined as Mg ha^−1^.

### Genomic data

We genotyped 180 out of the 202 DH lines, as well as the two parents, Tapidor and Ningyou7, using a 60,000 SNP array (Zhang et al., 2016). After quality control, 13,753 SNP markers remained, which were polymorphic, with missing values <5% and a minor allele frequency (MAF) >5%. We further removed SNPs in a perfect linkage disequilibrium that resulted in 1,527 unique SNPs (Zou et al., 2016). The genotypes of the 318 TNRC-F_2_ hybrids (Supplementary Figure 1) were inferred based on the genotypes of their respective parents. For the independent validation population with 37 new single crosses, we also genotyped the 18 new parental lines using the same SNP array and SNP marker filter parameters to get the genotypes of the 37 crosses based on the genotypes of the parents.

### Phenotypic data analyses

All quantitative genetic parameters were estimated based on the performance of 318 hybrids and the 180 DH parents. After outlier tests (Anscombe and Tukey, 1963), the adjusted means of the genotypes within each environment were estimated based on the following mixed model:

$$y_{in}=\mu +g_{i}+\varepsilon_{in},$$

where *y*_*in*_ was the seed dry weight of the *n*th observation for the *i*th genotype, μ was the mean value, *g*_*i*_ was the genetic value of the *i*th genotype, and ε_*in*_ was the corresponding residual. The mean value and genetic value were modeled as fixed effects, and the residual was treated as a random effect.

The adjusted means of seed dry weight for each genotype across environments were estimated with the following model:

$$y_{ik}=\mu +g_{i}+l_{k}+\varepsilon_{ik},$$

where *y*_*ik*_ was the adjusted mean of the *i*th genotype in the *k*th environment, μ was the overall mean, *g*_*i*_ was the genetic value of the *i*th genotype, *l*_*k*_ was the effect of the *k*th environment confounded with replicate effects, and ε_*ik*_ was the residual. The mean and genetic values were modeled as fixed effects, environment effects, and residuals were treated as random effects.

In addition, we estimated the genetic variance components of hybrids and parental lines, as well as the variance of genotype × environment interactions using a one-step model:

$$\begin{array}{l} y_{ijkn}=\mu +a+p_{ij}+{\left(pl\right)}_{ijk}+m_{i}+f_{j}+s_{ij}+{\left(ml\right)}_{ik}+{\left(fl\right)}_{jk}+\\ \;{\left(sl\right)}_{ijk}+l_{k}+e_{ijkn}, \end{array}$$

where *y*_*ijkn*_ was the phenotypic performance of the *n*th observation for the *ij*th entry (line *i* = *j*, or hybrid *i* ≠ *j*) in the *k*th environment, μ was the overall mean, *a* was the group effect for lines and hybrids, *p*_*ij*_ was the genetic effect of the parental lines, *(pl)*_*ijk*_ was the interaction effect of the *ij*th parental line with the *k*th environment, *m*_*i*_ was the GCA effect of the *i*th male line, *f*_*j*_ was the GCA effect of the *j*th female line, *s*_*ij*_ was the SCA effect of crosses between lines *i* and *j, (ml)*_*ik*_ and *(fl)*_*jk*_ were GCA × environment effects of female and male lines, and *(sl)*_*ijk*_ was SCA × environment interaction effects, *l*_*k*_ was the effect of the *k*th environment, and *e*_*ijkn*_ was the residual. All effects were treated as random effects, except for the mean value and group effects.

The broad-sense heritability was calculated as the ratio of genotypic to phenotypic variance:

$$H^{2}=\;\frac{\sigma_{G}^{2}}{\sigma_{G}^{2}+\frac{\sigma_{G\times E}^{2}}{N_{E}}+\frac{\sigma_{E}^{2}}{{N_{E}}^{*}N_{R}}},$$

where *N*_*E*_ referred to the number of environments, *N*_*R*_ was the average number of replications per environment, $\sigma_{G}^{2}$ was the genotypic variance, $\sigma_{G\times E}^{2}$ was the variance of genotype multiplied by environment interaction, and $\sigma_{E}^{2}$ referred to the error variance.

### Genome-wide QTL mapping

Design matrices for additive and dominance effects were specified for the hybrids and their parental lines according to the F_∞_ metric (Falconer and Mackay, 1996). Data from each environment were used in QTL mapping. To correct for potential population stratification, the one minus the Rogers' distance matrix was used as a kinship matrix in the genome-wide QTL mapping scan (Zhao et al., 2013). Genome-wide scans for marker-trait associations were conducted to detect main-effect QTL, as well as all first-order epistasis effect QTL.

For main effect QTL, the model was defined as the following Yu et al. (2006):

Y=Xβ+Ss+Zu+e.

*Y* stands for the adjusted entry means of the 498 genotypes, i.e., 180 DH lines and 318 TNRC-F_2_ hybrids, of each environment, β was a vector of environment effects, *s* was a vector of SNP effects, *u* was a vector of polygene background effects, and *e* was a vector of residual effects. *X, S*, and *Z* were incidence matrices relating *Y* to β, *s*, and *u*. β and *s* were treated as fixed effects, while *u* and *e* were treated as random effects. A Quantile-Quantile plot was used to test for proper control of population stratification (Yu et al., 2006). The Bonferroni–Holm procedure (Holm, 1979) was applied to correct for multiple tests at a significance level of *P* < 0.1.

We performed a full two-dimensional scan to detect epistatic QTL using the following model:

$$\begin{array}{l} Y=X\beta +Z_{A1}a_{1}+Z_{A2}a_{2}+Z_{D1}d_{1}+Z_{D2}d_{2}+Z_{AA}i_{11}\\ \;+Z_{A1D2}i_{12}+Z_{A2D1}i_{21}+Z_{DD}i_{22}+Zu+e, \end{array}$$

where *a*_1_, *a*_2_, *d*_1_, and *d*_2_, are additive and dominance effects of the two loci and *i*_11_, *i*_12_, *i*_21_, and *i*_22_ correspond to all four different epistatic interaction effects. *Z*_*A*1_, *Z*_*A*2_, *Z*_*D*1_, *Z*_*D*2_, *Z*_*AA*_, *Z*_*A*1*D*2_, *Z*_*A*2*D*1_, and *Z*_*DD*_ were incidence matrices for the effects defined above. In this model, all effects were treated as fixed effects except for *u* and *e*, which were treated as random effects. The epistasis model was implemented using the efficient mixed-model association (EMMA) approach, which significantly reduced the computation time (Kang et al., 2008). A permutation analysis with 1,000 repetitions was employed to correct for multiple testing of epistatic effects at a significance level *P* < 0.05 (Churchill and Doerge, 1994). The proportion of the phenotypic variance explained by single QTL was estimated using multiple regression with QTL ordered according to their *P*-values (Utz et al., 2000). The proportion of explained genotypic variance was determined as a proportion of explained phenotypic variance standardized by broad-sense heritability.

We applied five-fold cross validation to evaluate the accuracy to predict the genotypic values from the marker effects. The data set was randomly divided into an estimation set (100% of inbred lines and 80% of the hybrids) and a test set (20% of the remaining hybrids). QTL detection and estimation of marker effects were performed in the estimation set. The estimated marker effects were then used to predict the performance of the genotypes in the test set. The prediction accuracy was estimated as a Pearson correlation coefficient between predicted and observed values standardized with the square root of the heritability.

### Genome-wide prediction

Based on the adjusted entry means of the 498 genotypes, we applied genomic best linear unbiased prediction (Vanraden, 2008; Zhao et al., 2015a) that considered additive, dominance, and epistatic effects (Zhao et al., 2015a). The model including only additive effects was:

$$y=1_{n}\mu +g_{a}+e.$$

The model including additive and dominance effects was:

$$y=1_{n}\mu +g_{a}+g_{d}+e.$$

*Y* were the adjusted entry means of the 498 genotypes across the three environments, 1_*n*_ was a vector of ones and *n* was the number of genotypes, μ referred to the overall mean across all three environments, and *g*_*a*_ and *g*_*d*_ represented the additive and dominance effects, respectively. In the model, μ was a fixed effect, and the remaining effects were all random effects following normal distributions $g_{a}\sim N\left(0,G_{a}\sigma_{a}^{2}\right)$, $g_{d}\sim N\left(0,G_{d}\sigma_{d}^{2}\right)$, and $e\sim N\left(0,I\sigma_{e}^{2}\right)$, where *G*_*a*_ and *G*_*d*_, were the relationship matrices corresponding to additive and dominance genetic effects, and $\sigma_{a}^{2}$, $\sigma_{d}^{2},$ and $\sigma_{e}^{2}$ were the variance of additive effects, dominance effects, and the residuals. Details on the implementation of these relationship matrices can be found in Zhao et al. (2015a). We used a shrinkage method to calculate the relationship matrices (Ledoit and Wolf, 2004; Endelman and Jannink, 2012). All of the above GBLUP models were implemented using the R package BGLR (Perez and Campos, 2014). In addition, we employed a G-BLUP approach using a Gaussian kernel to evaluate the relevance of epistasis for prediction accuracies. The Gaussian kernel method has the benefit of considering all higher-order epistatic interact effects. The G-BLUP model using the Gaussian kernel was implemented by using the R package rrBLUP (Endelman, 2011). The prediction accuracies were evaluated using the cross-validation scenarios outlined above. We also evaluated the prediction accuracy for the independent validation set using the same training set.

## Results

### Phenotypic data analyses of seed yield

The Best Linear Unbiased Estimations of seed yield for the single environments were significantly (*P* < 0.01) correlated, with Pearson moment correlation coefficients ranging from 0.48 to 0.59 (Supplementary Figure 2). The analyses across environments revealed genetic variance components, which were significantly (*P* < 0.05) larger than zero (Table 1). The broad-sense heritability estimates were 0.47 for the TNRC-F_2_ hybrid population and 0.48 for the TNDH parental population. We further decomposed the genetic variance for seed yield of the TNRC-F_2_ hybrid population into variances due to general ($\sigma_{\text{GCA}}^{2}$) and specific combining ability effects ($\sigma_{\text{SCA}}^{2}$). The $\sigma_{\text{SCA}}^{2}$ was 1.4 times larger than $\sigma_{\text{GCA}}^{2}$, and the variance of interaction effects between environments and SCA effects $\sigma_{\text{Environment}\times \text{SCA}}^{2}$ was 2.3 times larger than $\sigma_{\text{Environment}\times \text{GCA}}^{2}$.

**Table 1:** **Estimates of variance components (σ^2^) and broad-sense heritability of 318 hybrids and 180 parents evaluated for seed yield (Mg ha^−1^) across three environments**.

**Source**	**Hybrids**	**Parents**
$\sigma_{\text{Genotype}}^{2}$	0.0267^***^	0.0266^***^
$\sigma_{\text{GCA}}^{2}$	0.0110^*^	–
$\sigma_{\text{SCA}}^{2}$	0.0156^**^	–
$\sigma_{\text{Genotype}\times \text{Environment}}^{2}$	0.0805^***^	0.0770^***^
$\sigma_{\text{Environment}\times \text{GC}\text{A}}^{2}$^a^	0.0243^***^	–
$\sigma_{\text{Environment}\times \text{SC}\text{A}}^{2}$^b^	0.0561^***^	–
$\sigma_{\text{Residual}}^{2}$	0.0211	0.0211
Heritability	0.47	0.48

*, **, and *** *means significantly different from zero at P < 0.1, P < 0.01, and P < 0.001, respectively*.

a *General combining ability effects*.

b *Specific combining ability effects*.

**Figure 1:**
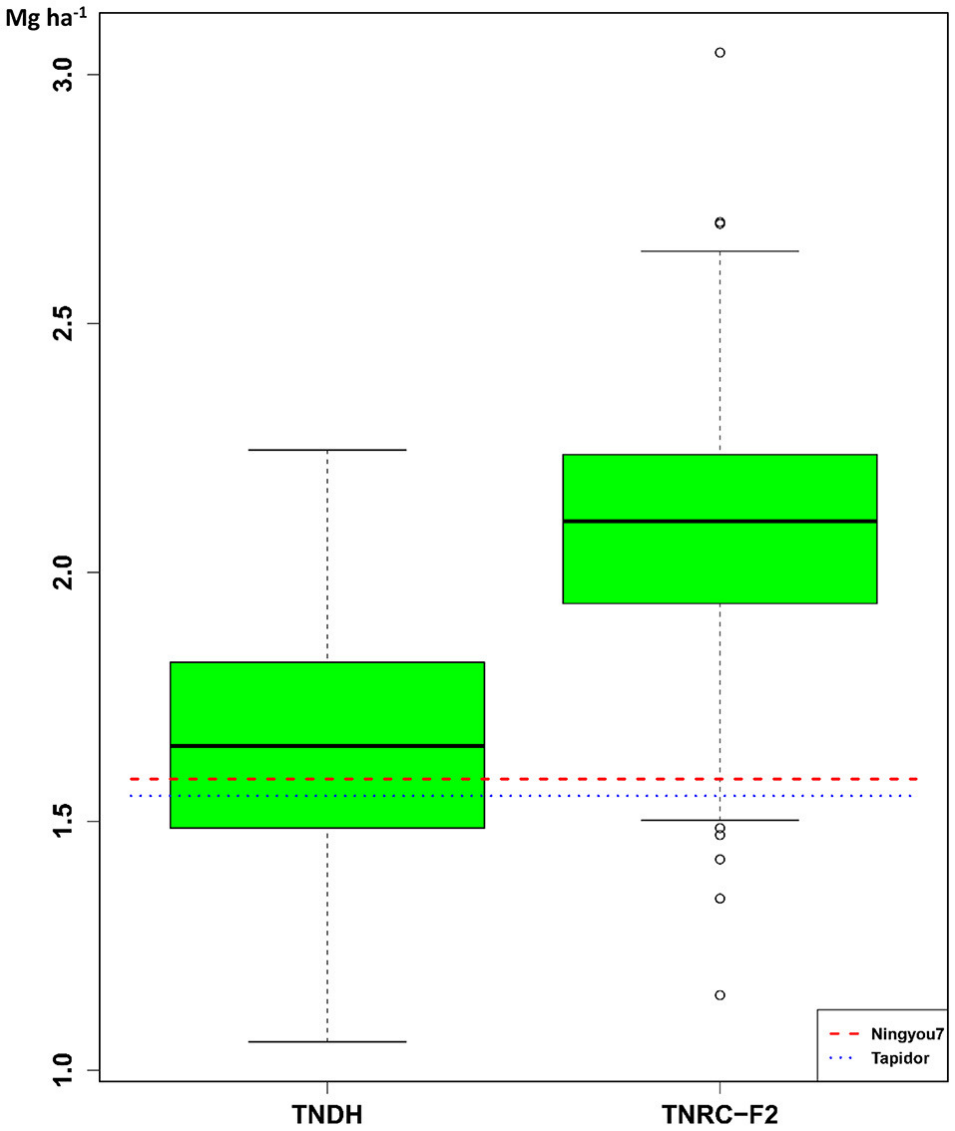
**Box-and-Whisker plots of the distribution of Best Linear Unbiased Estimations (BLUEs) for seed yield of the 180 DH lines (TNDH) and the 318 single-cross hybrids (TNRC-F_2_)**. The horizontal lines refer to the performance of the founder parents: Tapidor and Ningyou7.

Substantial transgressive variation was observed when comparing the seed yield of the TNDH parental population with the performance of the two founder lines, Tapidor and Ningyou7 (Figure 1). The average seed yield of the 318 single-cross hybrids of the TNRC-F_2_ population was 2.09 Mg ha^−1^ and was 1.27 times larger than the average seed yield of the 180 lines of the TNDH parental population.

### Genome-wide QTL mapping

**Figure 2:**
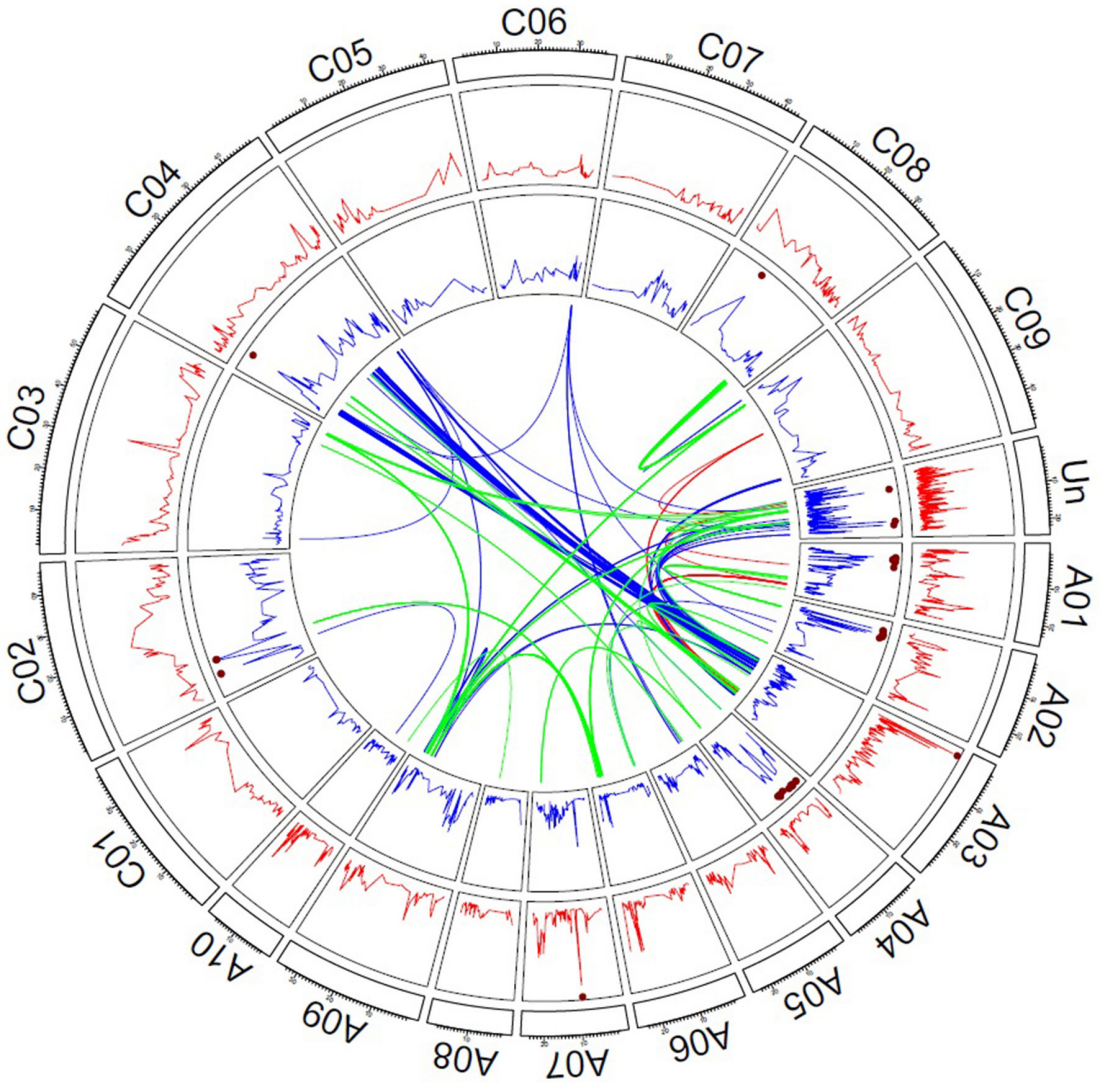
**Genetic architecture of hybrid performance in rapeseed**. The red lines refer to the -log_10_ (*P*-values) of the additive effects, and the blue lines refer to the -log_10_ (*P*-values) of the dominance effects. The brown dots mark the significant effects of *P* < 0.1 adjusted to apply the Bonferroni–Holm procedure (Holm, 1979). Links in the center of the circle represent significant digenic interactions between SNP markers; red lines reflect additive-by-additive, blue lines additive-by-dominance, and green lines dominance-by-dominance interactions.

Genome-wide QTL mapping was performed in the combined TNRC-F_2_ hybrid and TNDH parental population. The Quantile-Quantile plots for the additive and dominance effects revealed that population stratification was properly controlled with a model including the kinship matrix (Supplementary Figure 3). In the genome-wide QTL mapping scan, there were 2 SNPs located on chromosomes A03 and A07, which significantly contributed to the additive genetic variation for seed yield (Figure 2). The SNP located on chromosome A03 explained 7% and the SNP on chromosome A07 explained 4% of the total genotypic variance (Supplementary Table 2). Moreover, 32 SNPs exhibited significant dominance effects and were mainly located on chromosomes A01, A02, A04, and C02. The SNPs with significant dominance effects explained 44% of the genotypic variance.

A full two-dimensional scan for epistatic effects was performed, which revealed 18 significant additive by additive effects, 129 significant additive by dominant effects, and 104 significant dominance by dominance effects (Figure 2). The epistatic interactions mainly involved SNPs, which were located on chromosome A03, A09, and C04. All 251 epistatic effects explained 31% of genetic total genetic variance (Supplementary Table 3).

Five-fold cross-validation was performed for the genome-wide QTL mapping study to obtain unbiased estimates of the total genetic variation explained by additive, dominance, and epistatic effects. The prediction accuracy was measured as a Pearson moment correlation between predicted and observed values of the test population standardized with the square root of the broad-sense heritability. The prediction accuracy of additive effects amounted to 0.18 and increased to 0.26 when modeling additional dominance effects. Adding epistatic interaction effects did not alter the prediction accuracy of the hybrid performance.

### Genome-wide prediction of seed yield evaluated by applying cross validations

**Figure 3:**
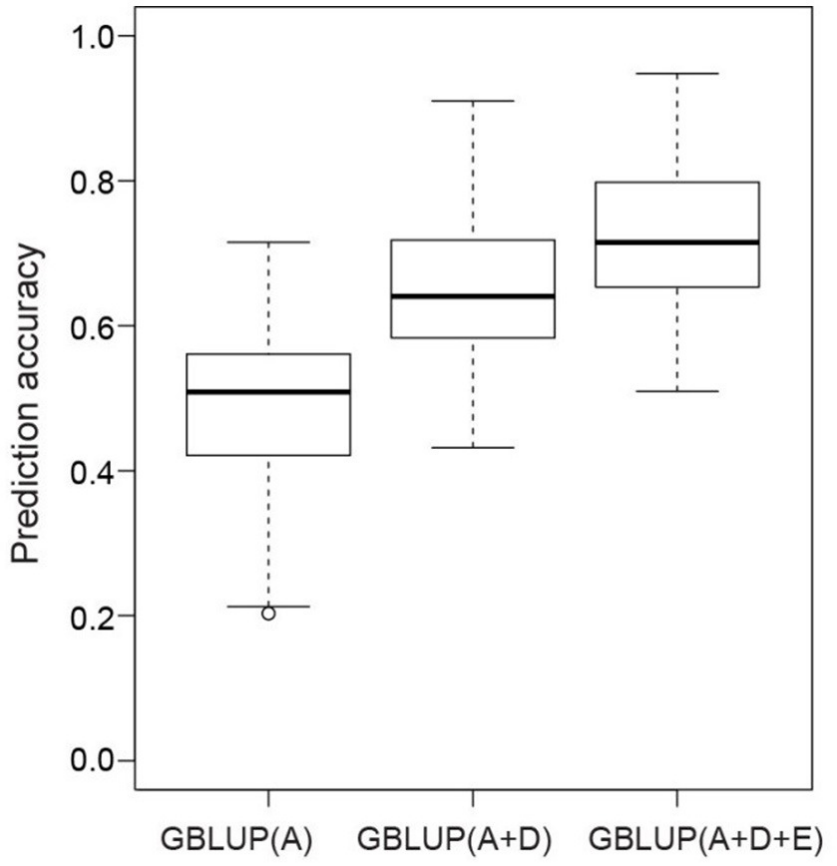
**Box-Whisker plots of whole-genome best linear unbiased prediction (GBLUP) accuracies of seed yield evaluated with five-fold cross validation**. Additive (A), dominance (D), and epistatic effects (E) were gradually included in the GBLUP models.

The accuracies of three different genome-wide prediction models were compared by applying five-fold cross validation (Figure 3). We observed an increase in the prediction accuracy from the model, which considered only additive effects (0.49) compared with the model with additive plus dominance effects (0.65) and with the highest accuracy of 0.72 observed when modeling additive, dominance, and epistatic effects. To summarize, the benefits were twice as much when including additionally dominance effects compared with epistatic effects.

### Genome-wide prediction of seed yield evaluated by applying independent validations

The accuracy of the prediction model developed based on the TNDH and TNRC-F_2_ populations was further evaluated in an independent validation. Therefore, an additional set of 37 RC-F_2_ single-cross hybrids were generated and evaluated at an independent environment. The prediction accuracy was assessed again by standardizing the Pearson moment correlation between predicted and observed values by the square root of the broad-sense heritability. The latter was estimated based on the variance components observed for the TNRC-F_2_ population. The prediction accuracy was high and amounted to 0.49 for the genome-wide prediction approach while also considering additive effects. Interestingly, the prediction accuracy dropped severely when additionally dominance (0.15) or epistatic effects (0.08) were included.

## Discussion

The immortalized TNRC-F_2_ population used in our study was initially designed to determine the genetic basis of midparent heterosis of hybrids between the European winter-type cultivar Tapidor and the Chinese semi-winter type cultivar Ningyou7 (Shi et al., 2011). Immortalized F_2_ populations exhibited an expected allele frequency of one-half, which maximized the variance of dominance effects (Falconer and Mackay, 1996) and enhanced the variance of epistatic effects (Mackay, 2014). Because dominance and epistatic effects determine midparent heterosis (Melchinger et al., 2008), its genetic basis can be studied thoroughly in immortalized F_2_ populations. The variance of additive effects is twice as high in doubled haploid populations compared with hybrid populations (Falconer and Mackay, 1996). Thus, an integrated analysis of doubled haploid and immortalized F_2_ populations represents a powerful approach for studying the contribution of additive, dominance, and epistatic effects to hybrid performance. This approach encouraged us to implement a strategy that involved reanalyzing seed yield data of the TNDH and TNRC-F_2_ populations (Shi et al., 2011) using the newly available high-throughput 60,000 SNP array (Zhang et al., 2016).

### Hybrid performance is substantially influenced by dominance effects

Distinguishing the variance of the hybrid performance into $\sigma_{\text{GCA }}^{2}$and $\sigma_{\text{SCA}}^{2}$ provides the first insights into the role of additive and dominance effects. Assuming there is an absence of epistasis, $\sigma_{\text{GCA }}^{2}$is determined for immortalized F_2_ populations through additive effects and $\sigma_{\text{SCA}}^{2}$ was controlled by dominance effects (Lynch and Walsh, 1998). Thus, the predominance of $\sigma_{\text{SCA}}^{2}$ was 1.4 times larger than $\sigma_{\text{GCA}}^{2}$ (Table 1) and clearly points to the relevance of dominance effects in the TNRC-F_2_ hybrid rapeseed population. Our findings agreed with previous results on grain yield reported for F_2_ populations in maize (Wolf et al., 2000) and rice (Li et al., 2010) generated using the North Carolina Design III (Comstock and Robinson, 1952). By contrast, studies using factorial mating designs based on rapeseed (Brandle and McVetty, 1989), maize (Parisseaux and Bernardo, 2004), wheat (Zhao et al., 2015a), or barley diversity panels (Philipp et al., 2016) observed that $\sigma_{\text{GCA}}^{2}$ is the main component of the variance of the hybrid performance. These discrepancies can be explained by allele frequencies substantially deviating from one-half in factorial crosses among diverse inbred lines, which severely impacts the ratio of $\sigma_{\text{GCA}}^{2}$ vs. $\sigma_{\text{SCA}}^{2}$ (Falconer and Mackay, 1996).

The findings of the genome-wide QTL mapping study (Supplementary Table 2) complement the picture of the relevance of dominance effects at the molecular level. We detected 16 times as many marker-trait associations that contributed to the dominance more than to the additive variation (Figure 2). The findings were in line with earlier studies reporting that dominance effects are key factors of heterosis in rapeseed (Radoev et al., 2008; Basunanda et al., 2010; Shi et al., 2011; Bu et al., 2015; Wen et al., 2015). Nevertheless, it is important to note that these results were not cross-validated, which was recommended to obtain an unbiased picture of the contribution of genetic effects to phenotypic variation (Utz et al., 2000). When we applied cross validation, the variance explained by all main effect QTL decreased from 55 to 7%, but the advantage for prediction accuracy when fitting data besides the additive, as well as the dominance effects, remains substantial (44%).

The small proportion of genetic variance explained by all QTL indicates a complex genetic architecture of seed yield in rapeseed, with the presence of many QTL each contributing only a little to the phenotypic variation. Genome-wide predictions are more suitable to tackle such complex traits (Riedelsheimer et al., 2012). Similarly, we observed a 2.5 times higher accuracy for the genome-wide predictions based on main effects (Figure 3) compared with the approach using QTL only. Nevertheless, the benefits of modeling the effects of the additive along with the dominance effects was comparable (33%, Figure 3) to the results observed for QTL mapping (44%). In summary, the phenotypic data analyses, the QTL mapping, and the genome-wide prediction study suggested that dominance effects were substantially contributing to the phenotypic variation of seed yield for the Tapidor × Ningyou7 rapeseed hybrid.

### Genome-wide prediction revealed a prominent role of epistasis contributing to the hybrid performance

According to previous results (Radoev et al., 2008; Basunanda et al., 2010; Shi et al., 2011; Bu et al., 2015; Wen et al., 2015), a large number of 251 significant digenic epistatic effects were detected in the genome-wide QTL mapping study (Supplementary Table 3). Around 31.5% (79) of the digenic interaction were additive by dominance effects involving SNPs on chromosome A03 and C04 (Supplementary Table 3). These 79 epistatic pairs trace back to eight clusters of markers, five on chromosome A03, and three on chromosome C04. Some of the SNPs of the eight clusters were also associated with main effect QTL for flowering time, seed weight, seed number, and oil content (Supplementary Table 3). Marker Bn-scaff_16534_1-p2320270 located on C04 showed significant dominance effects (Supplementary Table 2) and was together with closely linked markers involved in 26 of the epistatic interactions between chromosomes A03 and C04. Thus, Bn-scaff165341-p2320270 is an interesting candidate for further fine mapping studies.

Despite the large number of 251 significant digenic epistatic effects, the cross-validated prediction accuracy did not increase when modeling epistatic effects beyond main effects. This can either point to the irrelevance of epistasis for seed yield in rapeseed or can be explained by the challenge to detect epistatic effects for the complex trait seed yield (Mackay, 2014). The results of the genome-wide prediction study support the latter explanation. The prediction accuracy increased by 11% compared with the prediction approach based on additive and dominance effects only (Figure 3). The observed increase is much higher than that reported in previous studies on genome-wide prediction of hybrid performance. Zhao et al. (2015a) observed a 2% increase in prediction ability when modeling epistatic effects in a large population of 1,604 wheat hybrids and their 135 parental inbred lines. The lower benefit observed for wheat vs. rapeseed can be explained by the more marginal allele frequencies in factorial mating designs compared with immortalized F_2_ design. Surprisingly, Xu et al. (2014) observed no benefits when modeling epistatic effects in a large immortalized F_2_ population comprised of 240 rice inbred lines and 360 F_2_ genotypes. The discrepancy between our findings and that of Xu et al. (2014) might point to differences in the genetic architecture among crop species, as has been observed for the genetic basis of heterosis in the self-pollinating species rice vs. the outcrossing species of maize (Garcia et al., 2008) and deserves further research.

The total population was used to estimate the genetic components of variance with a Bayesian generalized linear regression based on the design matrices of additive, dominance, and all types of digenic epistatic effects. We observed a prominent role of the variance of dominance effects contributing to 49% of the genetic variance (Supplementary Figure 4). Additive (27%) and the sum of all digenic epistatic effects (24%) contributed nearly equally to the genetic variance of the hybrid performance. This suggests that the Tapidor × Ningyou7 rapeseed hybrid successfully exploits all types of genetic effects. Whether our findings are also valid for further rapeseed hybrids or not, deserve further research in other genetic backgrounds.

### Additive effects were less affected by environment interactions than non-additive effects

The phenotypic data analyses revealed that $\sigma_{\text{GCA }}^{2}$ was less affected by varying environmental conditions than $\sigma_{\text{SCA}}^{2}$ (Table 1). This result contrasted with previous findings based on an F_2_ population derived from the B73 × Mo17 maize hybrid (Wolf et al., 2000), which again points to differences in the genetic architecture among crop species. Validating the developed prediction model in an independent sample of genotypes and environments revealed that the observation at the phenotypic level was also reflected at the molecular level. The prediction accuracy is stable for additive effects (0.49) but collapsed when adding dominance (0.15) or epistatic effects (0.08) (Figure 3). Thus, the development of robust hybrid prediction models, including dominance and epistatic effects, requires a much deeper phenotyping analysis in multi-environmental trials. The flip side of our finding is that prediction models focusing exclusively on additive effects yields already stable and high prediction accuracies. Additive models can be easier implemented simplifying the application of hybrid prediction in plant breeding programs.

## Conclusions

Our study was based on a doubled haploid and immortalized F_2_ population derived from the single-cross hybrid Tapidor × Ningyou7, and the conclusions are restricted to this gene space. The phenotypic data analyses, the QTL mapping, and the genome-wide predictions revealed that hybrid performance is driven by a mix of additive, dominance, and epistatic effects. Prediction accuracies substantially profited when integrating dominance and epistatic effects, which is most likely to result from using a mapping population with expected allele frequencies of one-half. Transferring the results to a broader diversity involves the challenge that this entails to move to a gene space with less balanced allele frequencies, which led to a low power to exploit dominance and epistatic effects. Further research is required to search for an optimum compromise between the inference space of the results and precision to predict additive as well as dominance and epistatic effects.

## Author contributions

PL and YZ performed the research, analyzed the data, and wrote the manuscript; GL, MW, DH, and JH analyzed the data; MW helped the genotyping and phenotyping of new hybrids; JM provided plant materials, the previous published data and suggestions; JZ and JR designed the research, analyzed the data, and wrote the manuscript. All authors revised, read, and approved the final manuscript.

### Conflict of interest statement

The authors declare that the research was conducted in the absence of any commercial or financial relationships that could be construed as a potential conflict of interest.

## Supplementary material

Supplementary Table 1 **The environments used for seed yield field trial of TNRC-F_2_ population**.

Supplementary Table 2 **Significant (Bonferroni–Holm significant level *P* < 0.1) marker-trait associations and the proportion of explained genotypic variance (GVE) detected in a genome-wide association mapping approach for seed yield**.

Supplementary Table 3 **Significant (Bonferroni–Holm significant level *P* < 0.1) epistasis interaction the proportion of explained genotypic variance (GVE) detected in a genome-wide-association mapping approach for seed yield**.

Supplementary Figure 1 **The schematic diagram of the crossing design for 318 TN RC-F_2_ hybrids used in this study**. The corresponding DH parents involved in each of the crossings are show on the x-axis and y-axis, respectively. The red boxes indicate the crosses.

Supplementary Figure 2 **The distribution and correlation among best linear unbiased estimates (BLUEs) of each environment**.

Supplementary Figure 3 **Quantile-Quantile plots of association mapping for determining the models of QTL detection for hybrid seed yield. (A,B)** showed the expected *P*-value of association mapping using different models for detecting the QTLs with additive effects and dominance effects, respectively. The blue and red lines showed *P*-values of association mapping using the model without kinship and with kinship, respectively.

Supplementary Figure 4 **Pie chart of genetic components of variance (additive variance $\sigma_{A}^{2}$, dominance variance $\sigma_{D}^{2}$, and respective epistatic variance components) estimated with Bayesian generalized linear regression**.
