# Supplementary material for: Hybrid Performance of an Immortalized F2 Rapeseed Population Is Driven by Additive, Dominance, and Epistatic Effects
Source: Front Plant Sci. 2017 May 18;8:815. doi: 10.3389/fpls.2017.00815 (PMC5435766; doi:10.3389/fpls.2017.00815)
Supplement: Supplementary Table 1 — The environments used for seed yield field trial of TNRC-F2 population. [file Table1.DOCX]

**Supplementary Table1: The environments used for seed yield field trial of TNRC-F_2_ population.**

| Environment* | Location and geographic feature | Rapeseed growing period |
| --- | --- | --- |
| S5 | Jiangling, E113^o^25'/N30^o^30'/40 m | Oct, 2004—May, 2005 |
| S6 | Daye, E114^o^48'/N30^o^06'/100 m | Oct, 2005—May, 2006 |
| N6 | Dali, E109^o^56'/N34^o^52'/800 m | Sep, 2005—Jun, 2006 |

^*The first letter represents the orientation of the location in China: Jiangling and Daye are in southern (S) China and Dali is in northern (N) China; the last letter represents the year of harvest (Shi et al., 2011).^
