# Supplementary figures and images for: Hybrid Performance of an Immortalized F2 Rapeseed Population Is Driven by Additive, Dominance, and Epistatic Effects
Source: Front Plant Sci. 2017 May 18;8:815. doi: 10.3389/fpls.2017.00815 (PMC5435766; doi:10.3389/fpls.2017.00815)

# Crossing Design

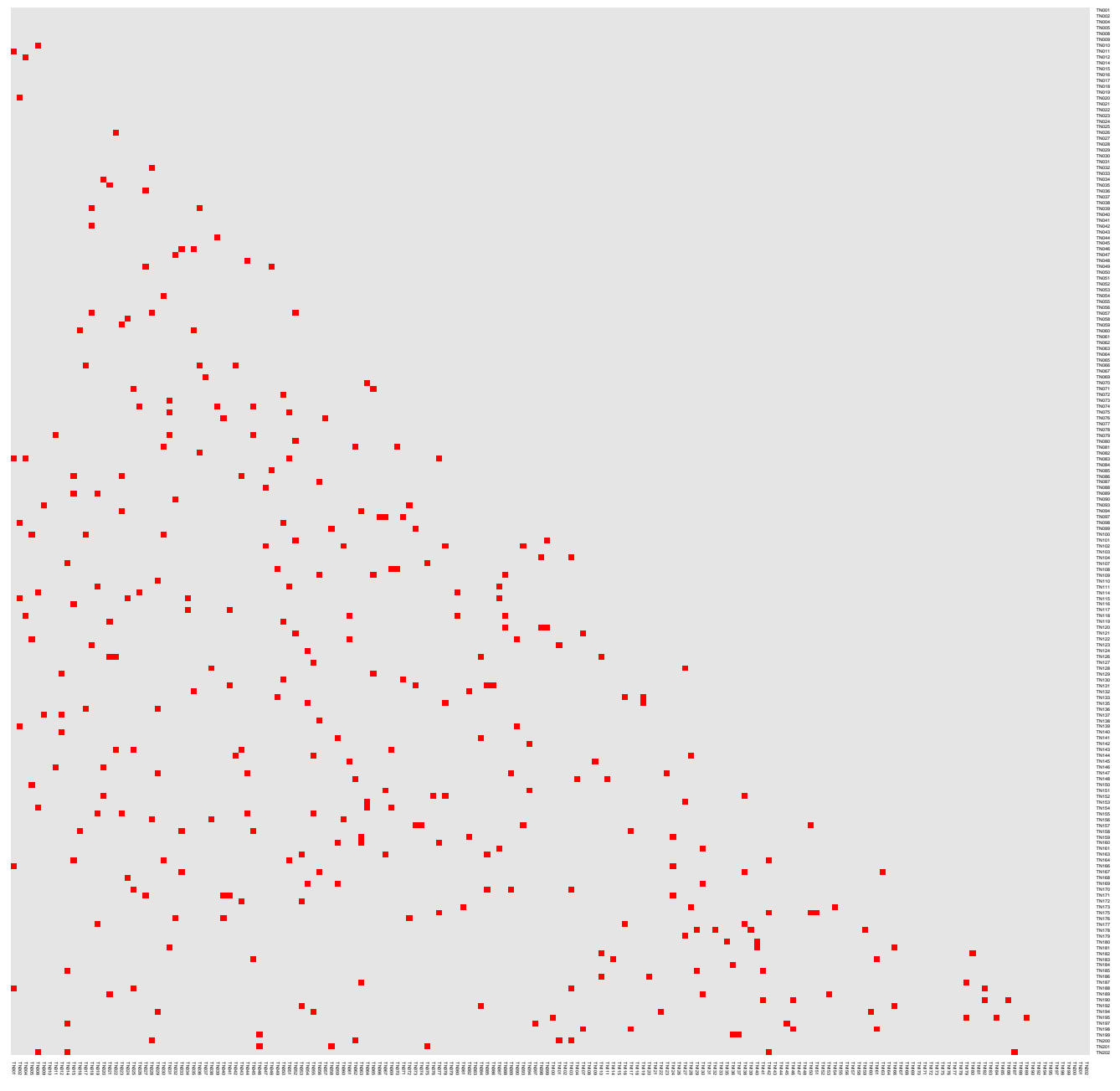

Supplement: Supplementary Figure 1 — The schematic diagram of the crossing design for 318 TN RC-F2 hybrids used in this study. The corresponding DH parents involved in each of the crossings are show on the x-axis and y-axis, respectively. The red boxes indicate the crosses. [file Image1.PDF]

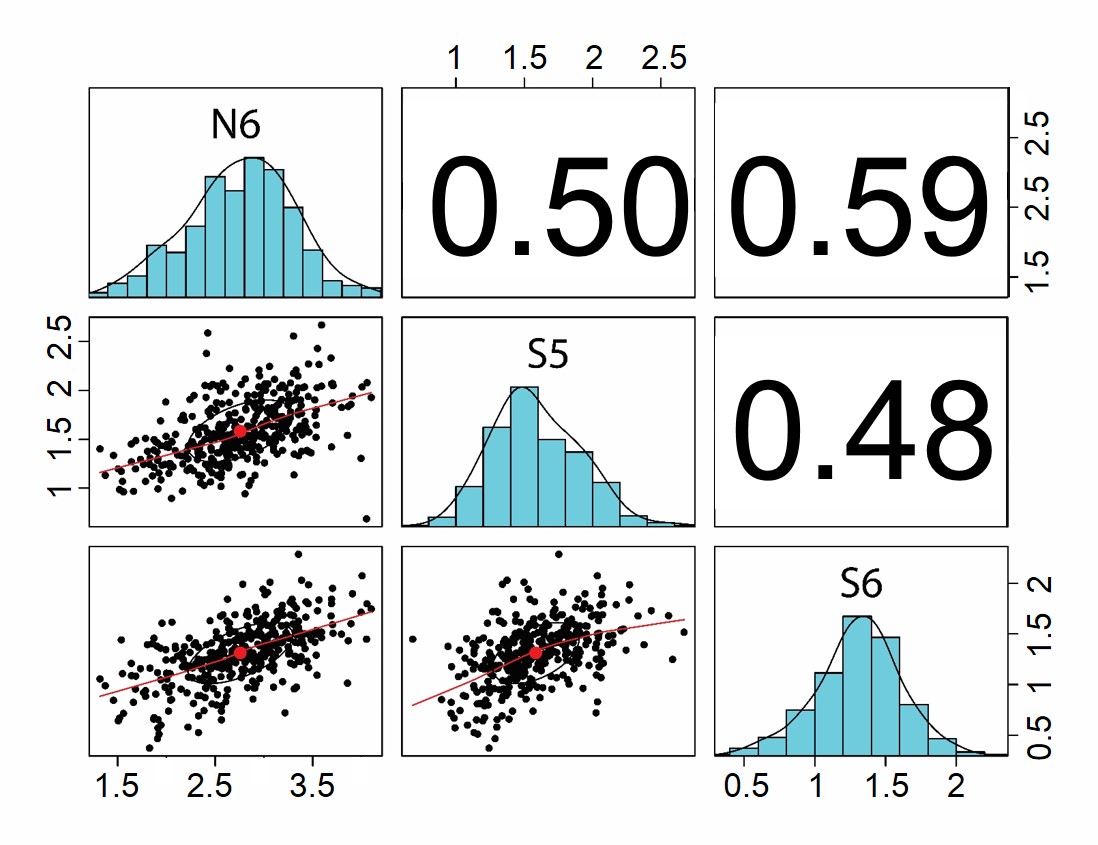

Supplement: Supplementary Figure 2 — The distribution and correlation among best linear unbiased estimates (BLUEs) of each environment. [file Image2.JPEG]

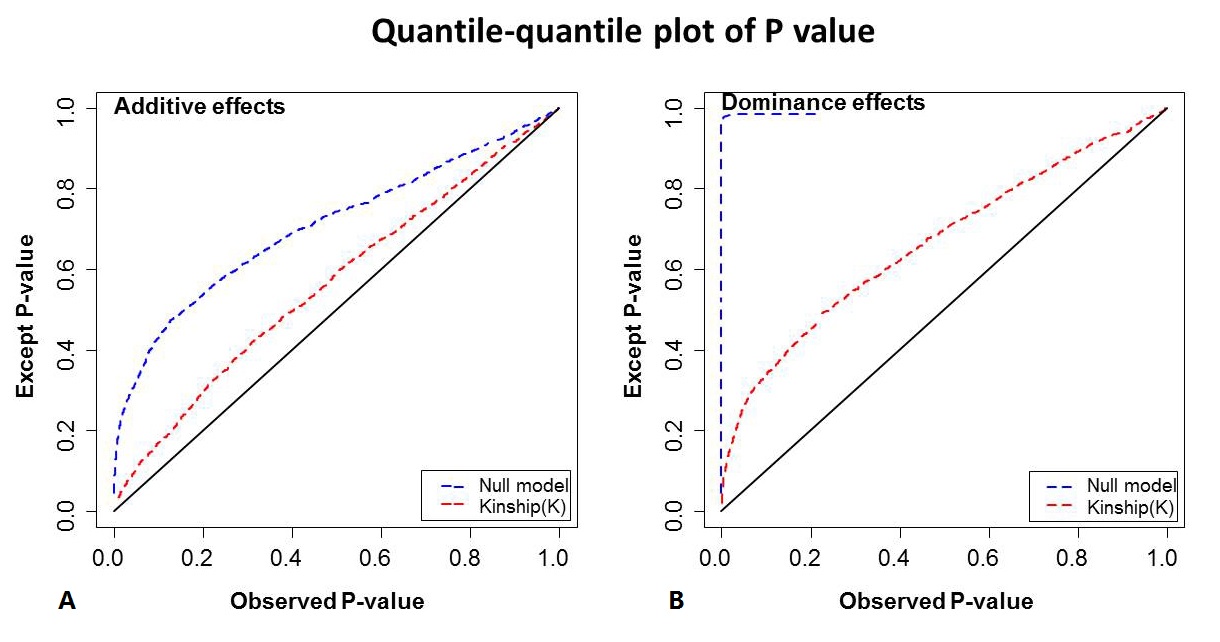

Supplement: Supplementary Figure 3 — Quantile-Quantile plots of association mapping for determining the models of QTL detection for hybrid seed yield. (A,B) showed the expected P-value of association mapping using different models for detecting the QTLs with additive effects and dominance effects, respectively. The blue and red lines showed P-values of association mapping using the model without kinship and with kinship, respectively. [file Image3.JPEG]

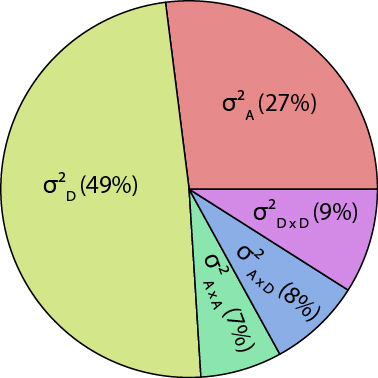

Supplement: Supplementary Figure 4 — Pie chart of genetic components of variance (additive variance σA2, dominance variance σD2, and respective epistatic variance components) estimated with Bayesian generalized linear regression. [file Image4.JPEG]
